# Forest fire detection and recognition method based on improved YOLOv5-ACE algorithm

**DOI:** 10.1371/journal.pone.0343592

**Published:** 2026-03-09

**Authors:** Yu Zhao, Chao Tang

**Affiliations:** School of Emergency Management, Chongqing Vocational Institute of Safety Technology, Wanzhou, China; G H Raisoni College of Engineering and Management, Pune, INDIA

## Abstract

Currently, forest fires have become a major fire safety issue. To detect forest fires and optimize the accuracy, a forest fire detection and recognition model based on an improved YOLOv5-ACE algorithm is proposed. In response to the difficulties of small target detection in forest fires, poor adaptability to complex backgrounds, and deployment limitations of edge devices, the CBAM and the ASPP multi-scale feature extraction module are introduced to enhance the ability of target feature capture and small target detection. The algorithm is lightweight by combining the grouped convolution of ShuffleNet v2 and the global dependency capture of ViT, while improving the positioning accuracy and anti-interference ability. Compared with the traditional YOLOv5, the detection accuracy has increased by 11.5%, ultimately reaching 92.3%, and the recall has increased by 6.8% to 91.6%. Through hypothesis testing, all performance improvements have statistical significance (*p <* 0.05). The proposed method can detect forest fires more quickly and accurately, which has good guiding significance for preventing the occurrence of forest fires.

## 1 Background

Forest fires are one of the major global disasters, posing a serious threat to ecosystems and socio-economic stability. As global climate change and human activities intensify, forest fires are becoming more frequent and widespread. Their rapid spread and difficult to control characteristics make early detection and identification a key link in reducing fire losses [1]. Traditional forest fire detection methods mainly rely on manual inspections, satellite remote sensing, and fixed monitoring stations. Nevertheless, these methods have drawbacks, including low efficiency, high false alarm rates, and insufficient ability to detect small target flames, making it difficult to meet the high-precision and real-time of modern forest fire prevention and control [2,3]. The rapid development of Unmanned Aerial Vehicle (UAV) technology and edge computing means that target detection algorithms depending on deep learning offer new ideas and methods for forest fire detection. UAV can quickly reach the fire scenario to obtain high-resolution image and video data, while edge computing can process the data in real-time, thereby minimizing data transmission latency [4]. As a single-stage object detection strategy, You Only Look Once (YOLO) has been extensively used in various object detection tasks. The existing YOLO variants have clearly failed in forest fire scenarios with highly dynamic smoke and flame patterns. Although the YOLO series models can achieve an accuracy of 96% in human body detection after disasters, in the dynamic scenario of forest fires, due to the complex terrain and the limitations of equipment computing power, feature confusion is prone to occur when identifying flame targets in dynamic smoke occlusion areas, and the false detection rate is 21.6% higher than that in static scenarios [5]. In traditional YOLOv5 applications without customized annotation schemes, when faced with dynamic changes in flame patterns and interference from smoke diffusion, the missed detection rate of early tiny fire points is as high as 29.3%, which is significantly higher than the 98% fire event detection accuracy after optimized annotation. The core reason lies in the fact that its fixed anchor frame and feature extraction network are difficult to adapt to the irregular jitter of flame edges and the instantaneous expansion and contraction of smoke forms [6]. The essence of these failed cases is that the existing YOLO variants are mostly designed for general targets and do not fully consider the core characteristics of forest fires, such as the dynamic interweaving of smoke and flames, instantaneous changes in target scale, and complex background interference. This leads to a mismatch between their feature modeling capabilities and the adaptability to dynamic scenarios, making it difficult to meet the actual needs of rapid early fire detection.

With the popularization of UAV and edge computing, the improved YOLO-based fire detection method has become a focus. In response to the insufficient real-time recognition accuracy and high false alarm rate in smart city fires, F. M. Talaat et al. integrated deep learning YOLOv8, and used a three-layer architecture of fog-cloud-IoT to extract flame features in real-time. Experiments showed that this method achieved an accuracy of 97.1% across all categories and could be extended to forest fire monitoring scenarios, providing new ideas for improving the YOLOv5-ACE algorithm [7]. X. Jiang et al. proposed a detection strategy that combined deformable attention and lightweight feature extraction for early forest flame edge blurring and high false alarms in complex environments. A small target detector was introduced to capture weak fire points. The experiment showed that this strategy increased the average accuracy and comprehensive score by about 10%, significantly reducing false positives and false negatives, providing a new approach for early identification of forest fires [8]. In response to the high loss of details and false alarms in early recognition of forest fires, C. Chen et al. improved the multi-scale fusion strategy by introducing attention mechanisms between adjacent layers of the backbone network and supplementing them with Data Augmentation (DA) training. The flame detection method had an average accuracy of 86.87% and 85.66% in two test sets, respectively, with a false alarm rate reduced to less than 1% [9]. In response to the blurred edges of forest fire smoke screens and the strip flames to be missed, G. Nie et al. embedded snake-like convolution and depth aware neck structure into the detection backbone to construct a recognition framework that balanced local granularity and global semantics. They also introduced multi-angle Intersection over Union (IOU) ratio loss to optimize the bounding box. In public dataset validation, the average accuracy of this method reached 80.6%, combining real-time performance with high accuracy, providing a feasible solution for aerial monitoring of forest fires [10]. Aiming at the insufficient prediction accuracy of rice yield and difficulty in quantifying the fluctuation range, Lu Y et al. proposed a quantitative regression bidirectional long short-term memory network that integrated hyperspectral images and multi-phenotypic data. A multi-head self-attention mechanism was introduced to weight key growth factors, synchronously estimate the mean and interval. The test results showed that the coefficient of determination of this model reached 0.927, the average absolute percentage error was only 2.21%, and the root mean square error was 0.22 tons per hectare. It was significantly better than traditional methods and provided a reliable basis for food security decision-making [11]. Aiming at the insufficient fusion of local details and global context in the identification of five major diseases such as rice blast and sheath blight, Lu Y et al. proposed an improved mobile transform network, which extracted local features through lightweight mobile network branches and captured global relationships through windowed transform branches, and then achieved dual-representation fusion through channel cascading. The experimental results showed that the average recognition accuracy rate of rice diseases reached 99.62%, which was 0.42%−38.09% higher than that of other mainstream methods, respectively, providing a feasible solution for precise diagnosis of field diseases [12].

Most of the existing work focuses on optimizing a single module and has not yet filled the two core theoretical gaps: First, it only enhances local feature extraction and fails to address the feature modeling gap between local shape fluctuations and global spatial correlations of dynamic firepower targets. Secondly, lightweight design and adaptability to complex scenarios often show a mutually exclusive relationship, and there is a lack of a collaborative module architecture that can balance the two. This is precisely the direct incentive for the research to choose the combination of CBAM, ASPP, ShuffleNet v2 and ViT. Therefore, a forest fire detection method based on the Improved YOLOv5-ACE Algorithm (YOLOv5-ACE) is proposed. This method improves the detection ability for small target flames and adaptability to complex backgrounds by introducing Convolutional Block Attention Module (CBAM) and Atrous Spatial Pyramid Pooling (ASPP) module. ShuffleNet v2 and Vision Transformer (ViT) modules are introduced to achieve model lightweighting while maintaining high detection accuracy. In addition, the research aims to establish an image dataset by collecting multiple scenario fire images, filtering and annotating them, and expanding the dataset with various DA strategies to optimize the generalization ability and robustness. The innovation of the research lies in comprehensively applying multiple improvement strategies, which optimizes the detection accuracy, and significantly weakens the calculation cost, especially in the resource constrained edge computing environment. The contribution of the research lies in proposing a forest fire detection and recognition method. By introducing CBAM and ASPP modules, the detection ability for small target flames and adaptability to complex backgrounds are significantly improved. Furthermore, ShuffleNet v2 and ViT modules are used to achieve a lightweight design, which reduces computational costs while maintaining high detection accuracy. In addition, a dataset containing multiple scenario fire images is established, and various DA strategies are used to expand the dataset to improve generalization ability and robustness. These improvements enhance detection accuracy and efficiency, offering an efficient and reliable solution for forest fire monitoring in resource-constrained edge computing environments.

## 2 Fire detection and recognition based on improved YOLOv5-ACE algorithm

The research enhances the detection capability of small target flames and adaptability to complex backgrounds by introducing CBAM and ASPP modules. ShuffleNet v2 and ViT modules are adopted for lightweight design, which maintains high detection accuracy while reducing computational costs. The research also establishes image datasets. By collecting multiple scenario fire images and filtering and annotating them, combined with various DA strategies, rich and diverse data support is provided for model training to optimize the generalization ability and robustness.

### 2.1 Fire detection algorithm based on improved YOLOv5

**Fig 1:**
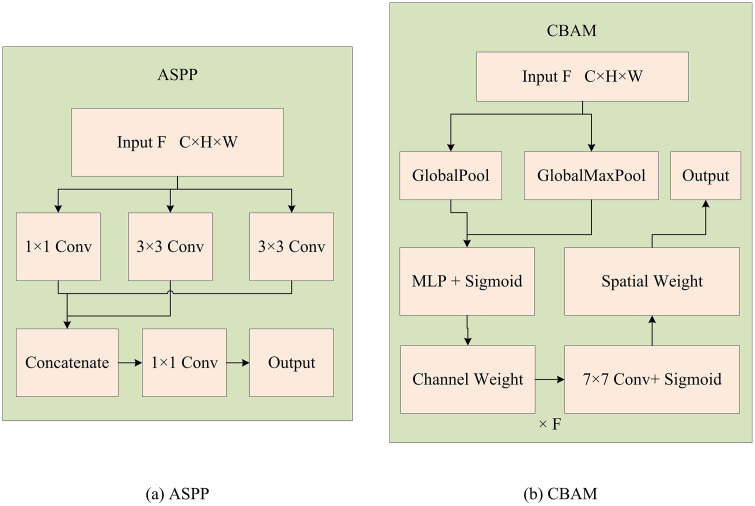
The structure of CBAM and ASPP modules.

YOLOv5 is a widely used and efficient single-stage object detection algorithm with advantages such as fast speed and high accuracy. However, YOLOv5 still has some shortcomings in handling forest fire detection, such as low detection accuracy for small target flames and poor adaptability to complex backgrounds. Therefore, a method for detecting forest fires based on an improved YOLOv5 is proposed, which optimizes the detection ability for small target flames and adaptability to complex backgrounds by introducing CBAM and ASPP modules. CBAM is a dual-attention module that sequentially performs channel and spatial attention enhancement: channel attention weights feature channels based on global max-pooling and average-pooling results to highlight flame-related feature responses, while spatial attention uses convolution to focus on flame spatial regions by fusing global pooling features. ASPP adopts multi-scale dilated convolutions with different dilation rates and global pooling to extract multi-scale target features, effectively capturing information from tiny fire points to large-scale combustion areas. Aiming at weak small-target flame features and large scale differences in forest fire scenarios, CBAM is improved to adapt to flame target characteristics: its spatial attention uses a 7 × 7 convolutional kernel, matching 62% of medium and small-scale flames in the dataset, balancing local detail capture and background distinction. ASPP optimizes the combination of 1 × 1 and 3 × 3 convolutions with dilation rates of 6, 12, and 18, specifically targeting the multi-scale distribution of forest fire targets to solve the missed detection of tiny ignition points and inaccurate large-scale flames. ASPP directly replaces the SPP module in the original YOLOv5 backbone, expanding the receptive field through multi-scale dilated convolution to adapt to flame scale differences. CBAM is embedded before the three-scale feature output of the Neck’s FPN + PAN fusion layer, associating with the 80 × 80 × 256 and 160 × 160 × 128 feature maps from the backbone. It first weights key feature channels through channel attention, then enhances flame region responses through spatial attention and cooperates with ASPP to improve small-target detection accuracy in complex backgrounds [13]. The module combination takes feature enhancement and lightweight collaboration as the core innovation logic. Its theoretical support stems from the dual-contradiction resolution requirements of dynamic fire target detection: One is the contradiction between the weak flame features of small targets and the interference of complex backgrounds. The other is the contradiction between the high-precision requirements of the model and the resource constraints of edge devices. From a theoretical perspective, based on the complementary theory of CNN local feature extraction and Transformer global modeling, the CBAM, relying on the attention mechanism theory, focuses on the key features of the flame through dual-weight distribution of channels and spaces, solving the problem that the features of small targets are easily submerged by the background [14]. The ASPP module employs the multi-scale receptive field expansion theory and adopts a combination of convolutional kernels with different dilation rates to adapt to the multi-scale target distribution ranging from tiny fire points to large-area burning, solving the detection problem caused by target scale differences in complex backgrounds [15]. From the practical perspective, the grouped convolution and channel rearrangement techniques of ShuffleNet v2 adopt computational complexity optimization theory, which compresses the number of model parameters while ensuring the effectiveness of feature extraction [16]. The patch sequence processing mechanism of ViT is based on global dependency modeling theory, capturing the spatial correlation features of flames and backgrounds. The two form a collaborative architecture of lightweight and global feature capture [17]. ShuffleNet v2 frees up hardware resources for the high computational overhead tasks of ViT, while ViT makes up for the deficiency of global feature capture caused by lightweighting, ultimately achieving a balance between detection performance and resource consumption. Fig 1 presents the structure of CBAM and ASPP.

In Fig 1 (a), CBAM performs global pooling and shares the input feature map with a Multi-layer Perceptron (MLP) to obtain channel weights, multiplies the original image back, and then performs a 7 × 7 convolution to obtain spatial weights, which are then weighted again before outputting. In Fig 1 (b), ASPP extracts multi-scale features using four parallel paths: 1 × 1 convolution, 3 × 3 convolution with different dilation rates, and global pooling. After concatenation, the features are fused and output through 1 × 1 convolution. The CAM involves reducing the dimensionality of the input feature map using global Max Pooling (MP) and Average Pooling (AP), resulting in two feature maps. The kernel size selection is based on the flame scale distribution characteristics of the dataset. The spatial attention of CBAM adopts a 7 × 7 convolutional kernel, which is suitable for small and medium-sized flame targets that account for 62% of the dataset, while considering the differences in local details and surrounding backgrounds. ASPP selects 1 × 1 and 3 × 3 convolution kernel combinations (with dilation rates of 6, 12, and 18, respectively) to match the multi-scale distribution of flames ranging from tiny ignition points to large-scale combustion, effectively capturing features of targets at different scales. Then, the channel weight is calculated through MLP, as shown in equation (1).


$$W_{c}=\sigma \left(MLP\left(F_{max})+MLP(F_{arg}\right)\right)$$ (1)

In equation (1), $F_{max}$ and $F_{arg}$ are two feature maps, representing the calculated channel weights. σ signifies the Sigmoid activation function to normalize weights to the [0,1] interval. The SAM conducts global MP and global AP on the output of the CAM, resulting in two feature maps ${F^{\prime }}_{max}$ and ${F^{\prime }}_{\text{avg}}$. Then, the spatial weights are calculated through a 7 × 7 convolution operation, as shown in equation (2).


$$W_{s}=\sigma \left(Conv\left({F^{\prime }}_{max}+{F^{\prime }}_{\text{avg}}\right)\right)$$ (2)

In equation (2), $W_{s}$ is the convolution operation used to calculate spatial weights. Conv stands for 7 × 7 convolution operation. ${F^{\prime }}_{max}$ and ${F^{\prime }}_{\text{avg}}$ respectively represent the two-dimensional feature maps of CAM output feature maps after global MP and AP. The channel and spatial weights first enhance the key feature channels through channel weighting, and then focus on the serial normalization effect of the flame region through spatial weighting. The normalization result of the channel weights constrains the response range of spatial attention, and the spatial weights further screen the effective features after channel enhancement to avoid the interference of redundant information. Feature fusion multiplies the outputs of CAM and SAM to obtain the final feature map, as displayed in equation (3).


$$F_{\text{out}}=F_{\text{in}}\times W_{c}\times W_{s}$$ (3)

In equation (3), $F_{\text{out}}$ signifies the output feature map. $F_{\text{in}}$ signifies the input feature map. The workflow of the ASPP is as follows: Multi-scale feature extraction uses dilated convolutions with various sampling rates to obtain multi-scale features from the input feature map, resulting in multiple feature maps $F_{\text{1}},F_{\text{2}},\ldots ,F_{n}$. Global pooling conducts global AP on the input feature map to get the global feature map $F_{\text{global}}$. Feature fusion concatenates the multi-scale feature map and the global feature map to obtain the fused feature map, as presented in equation (4).


$$F_{\text{fuse}}=\text{Concat}(F_{\text{1}},F_{\text{2}},\ldots ,F_{n},F_{\text{global}})$$ (4)

In equation (4), $F_{\text{fuse}}$ is the fused feature map. By adjusting the number of channels in the fused feature map through 1 × 1 convolution, the final feature map is presented in equation (5).


$$F_{\text{out}}=Conv_{1\times 1}\left(F_{\text{fuse}}\right)$$ (5)

**Fig 2:**
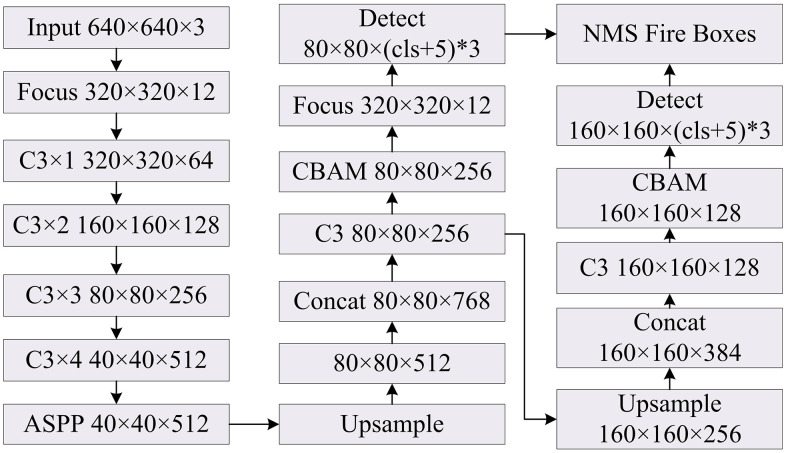
Structure of the improved YOLOv5 algorithm.

In equation (5), $F_{\text{out}}$ is the final feature map. Based on YOLOv5, CBAM and ASPP module are introduced to optimize the detection ability for small target flames and adaptability to complex backgrounds. Fig 2 presents the improved YOLOv5.

**Fig 3:**
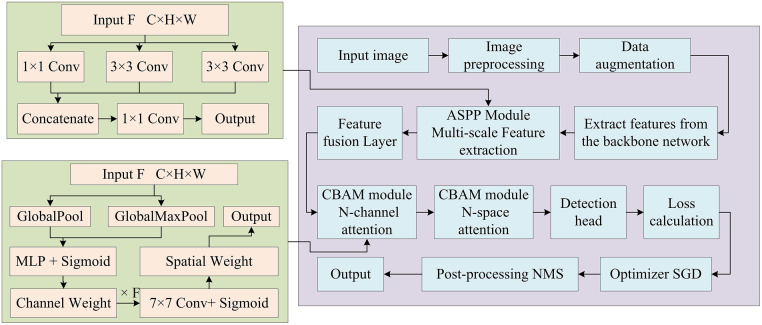
Fire detection process based on improved YOLOv5.

From Fig 2, the improved YOLOv5 algorithm inputs a 640 × 640 × 3 image and undergoes Focus slicing for dimensionality reduction. Backbone extracts 1/2, 1/4, 1/8, and 1/16 scale features layer-by-layer. Subsequently, ASPP is used to replace the original SPP to expand the receptive field. Neck takes a Bidirectional Feature Pyramid Network and Path Aggregation Network (FPN + PAN) for up-sampling and down-sampling fusion, and inserts the CBAM before the three scale outputs to enhance the target flame features. ASPP directly replaces the SPP module in the original YOLOv5 backbone network, expanding the perception range through multi-scale dilated convolution to accommodate the differences in fire sizes. In actual forest fire monitoring scenarios, such as unmanned aerial vehicle low-altitude patrols in northeastern coniferous forests of China and southwestern broad-leaved forests of China, CBAM is embedded before the three-scale feature output of the FPN + PAN fusion layer of the Neck, directly connecting with the two key feature maps output by the backbone network. One of them is an 80 × 80 × 256-sized feature map output by the C3 × 3 layer of the backbone network, mainly used to capture early small ignition points (pixel ratio < 5%) in the complex forest background, which are medium and small-scale fire features. The other is a 160 × 160 × 128-sized feature map obtained by upsampling and concatenation of the output features of the C3 × 2 layer of the backbone network, which is suitable for identifying the flame edges and smoke diffusion areas in open forest spaces. It first weights the key feature channels through the channel attention mechanism to suppress the interference from background features such as vegetation, rocks, and fog, and then enhances the response of the flame area through the spatial attention mechanism, and works collaboratively with ASPP to improve the detection accuracy of small target flames in complex backgrounds. Finally, the detection head performs parallel prediction and undergoes Non-Maximum Suppression (NMS) to obtain the result [18,19]. Fig 3 presents the fire detection process based on the improved YOLOv5.

From Fig 3, first, the input image is preprocessed and data enhanced, and then features are extracted through the backbone network. Next, the ASPP conducts multi-scale feature extraction, and the FPN layer performs feature fusion. Afterwards, the CBAM performs channel and spatial attention processing separately. The fused feature map is predicted using three detection heads of different scales, and the loss function is calculated using Extended IOU (EIOU). The optimizer Stochastic Gradient Descent (SGD) updates model parameters. The detection results are processed by NMS and finally output as a flame box for visual display [20,21]. Through the above improvements, the model can more effectively capture the features of small target flames when processing forest fire detection tasks, and maintain high detection accuracy even in complex backgrounds. The CBAM utilizes CAM and SAM to focus more on flame targets, thereby improving its ability to detect small target flames. The ASPP module expands the receptive field through multi-scale feature extraction to better adapt to flame targets of different sizes. The improved YOLOv5 maintains high performance, and also enhances the generalization ability, which can be better applied to different forest fire scenarios.

### 2.2 Lightweight design based on YOLOv5-ACE algorithm

**Fig 4:**
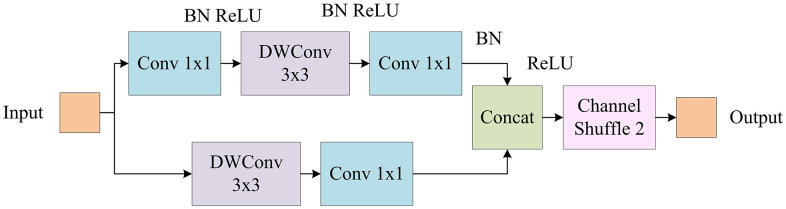
Structure diagram of ShuffleNet v2.

The research provides a detailed introduction to the forest fire detection method. By introducing the CBAM and ASPP modules, the detection ability for small target flames and adaptability to complex backgrounds are improved. However, although these improvements have improved detection performance, the complexity and computational cost of the model have also increased accordingly. To keep high performance while lowering computational cost, the research proposes a lightweight design based on the YOLOv5-ACE algorithm. This design achieves lightweighting while maintaining high detection accuracy by introducing ShuffleNet v2 and ViT modules. ShuffleNet v2 can improve model performance by reducing computation and memory usage. The core idea of ShuffleNet v2 is to reduce computational complexity while maintaining model performance through grouped convolution and channel rearrangement [22,23]. The structure of ShuffleNet v2 is illustrated in Fig 4.

**Fig 5:**
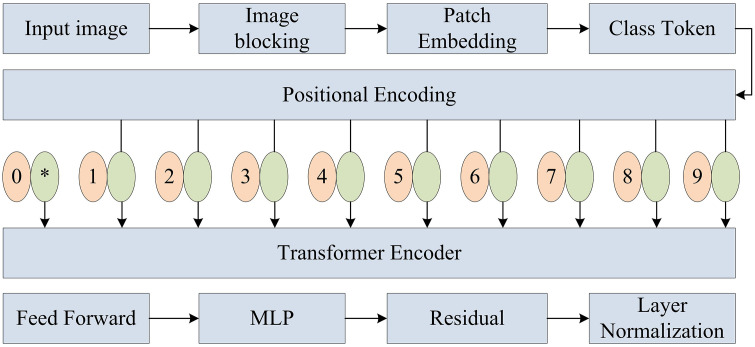
Structure of the ViT.

In Fig 4, ShuffleNet v2 goes through an initial Convolutional Layer (Conv) and pooling layer, and then enters four stages of grouped convolution and channel rearrangement operations, each stage containing two Convs. The feature map channel interaction is achieved through channel rearrangement operation in the middle. Each Conv is followed by a channel rearrangement operation to optimize the feature extraction efficiency. Finally, after passing through a Conv, a global pooling layer, and a fully connected layer, the classification result is output through the Softmax function. ViT is a Transformer-based image processing module that improves model performance by capturing global dependencies. ViT divides the image into multiple patches and inputs these patches as a sequence into Transformer for processing [24,25]. Fig 5 presents the structure of ViT.

In Fig 5, the input image of the ViT is first segmented into multiple patches. Each patch is mapped to a fixed dimensional space through linear transformation. Then, a special classification tag class token is added to these mapped patch sequences. Next, the positional encoding is assigned to the sequence to preserve spatial information. These sequences with positional information are fed into a Transformer encoder composed of multiple encoder layers, each of which includes a multi-head self-attention mechanism and a feedforward propagation network, as well as layer normalization and residual connections to stabilize the training process. After being processed by multiple layers of encoders, classification prediction is finally performed through an MLP and the Softmax function [26,27]. The multi-head attention is presented in equation (6).


$$\text{Attention}\left(Q,K,V\right)=\text{softmax}\frac{QK^{T}}{\sqrt{d_{k}}}V$$ (6)

In equation (6), Q, K, and V signify the query, key, and value. $d_{k}$ signifies the dimension of the key vector. The position encoding is shown in equation (7).


$$\left\{ \begin{array}{c} @c@\text{PosEncoding}\left(pos,2i\right)=sin\frac{pos}{10000^{2i/d\text{model}}}\\ \text{PosEncoding}(pos,2i+1)=cos\frac{pos}{10000^{2i/d\text{model}}} \end{array}\right.$$ (7)

**Fig 6:**
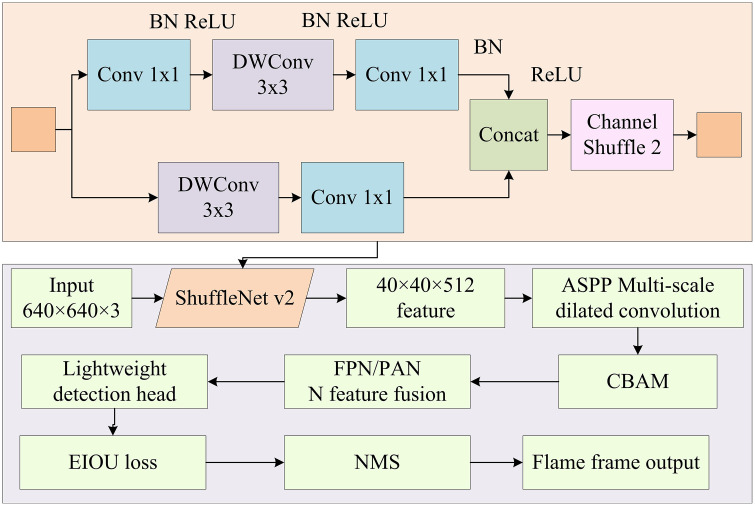
Structure diagram of YOLOv5-ACE.

In equation (7), pos signifies the position. i signifies the dimension. dmodel signifies the dimension of the model. The YOLOv5-ACE algorithm, based on the improved YOLOv5, further introduces ShuffleNet v2 and ViT modules to achieve lightweight design. Fig 6 presents the YOLOv5-ACE.

In Fig 6, the lightweight YOLOv5-ACE uses ShuffleNet v2 as the backbone, significantly reducing the parameter. The three-layer features output by the backbone are extended by ASPP to enhance the receptive field, and then uniformly strengthened by CBAM to enhance the flame channel and spatial weights. After lightweight FPN/PAN fusion, the flame bounding box is finally output by the three-scale detection head, balancing speed and accuracy. The ViT module captures global information by segmenting the image into multiple patches and inputting these patches as sequences into the Transformer for processing. The Complete IOU (CIOU) loss function is replaced with the EIOU loss function to further optimize detection accuracy [28,29]. In the task of locating boundary boxes for forest fires, the advantages of EIOU over CIOU have been verified through quantitative experiments. For targets with irregular flame edges and blurred smoke boundaries, the positioning error of EIOU is 12.7% lower than that of CIOU, and the bounding box regression speed is increased by 9.3%. Its design effectively decouples overlap loss, distance loss, and aspect ratio loss, solving the lag in aspect ratio optimization when CIOU locates small-scale flame targets. The IOU between the bounding box and the actual flame area increases by 8.9%. Equation (8) presents the EIOU loss function.


$$L_{\text{EIOU}}=1-\frac{\text{IOU}}{1+\frac{(w-\hat{w}{)}^{\text{2}}+(h-\hat{h}{)}^{\text{2}}}{w^{\text{2}}+h^{\text{2}}}+\frac{(x-\hat{x}{)}^{\text{2}}+(y-\hat{y}{)}^{\text{2}}}{w^{\text{2}}+h^{\text{2}}}}$$ (8)

In equation (8), (x,y,w,h) is the center coordinate and width/height of the real bounding box. $\left(\hat{x},\hat{y},\hat{w},\hat{h}\right)$ is the center coordinate and width/height of the predicted bounding box. Based on this lightweight design, the YOLOv5-ACE algorithm achieves significant improvements in detection accuracy, and performs well in computational efficiency and model size. ShuffleNet V2 reduces the parameter and computation required, which is suitable for resource-constrained edge computing devices. Meanwhile, the ViT further enhances the ability to capture whole information and improves its adaptability to complex backgrounds and small target flames. This design that combines CNN and Transformer architecture not only fully utilizes the advantages of both, but also further improves the performance through feature fusion. In addition, the EIOU loss function further optimizes the prediction accuracy of bounding boxes to more accurately locate flame targets in practical applications [30–32]. These improvements work together to make the YOLOv5-ACE algorithm perform well in forest fire detection tasks, providing strong support for building an efficient and robust intelligent monitoring system for forest fires.

### 2.3 Image data construction and augmentation

The study introduces a forest fire detection method based on the improved YOLOv5 algorithm and its lightweight version YOLOv5-ACE. These methods optimize the detection ability for small target flames and adaptability to complex backgrounds by introducing CBAM, ASPP module, ShuffleNet v2, and ViT module, while achieving lightweighting of the model. The performance largely depends on the quality and quantity of the training data. Therefore, the research focuses on establishing and enhancing image datasets. For forest fire detection tasks, fire images in actual scenarios are often affected by various factors such as lighting, occlusion, and background interference. This makes data collection and processing even more difficult. Therefore, the research not only needs to establish a dataset containing various fire scenarios and small target flames, but also needs to expand the dataset through DA strategies to improve the robustness.

**Fig 7:**
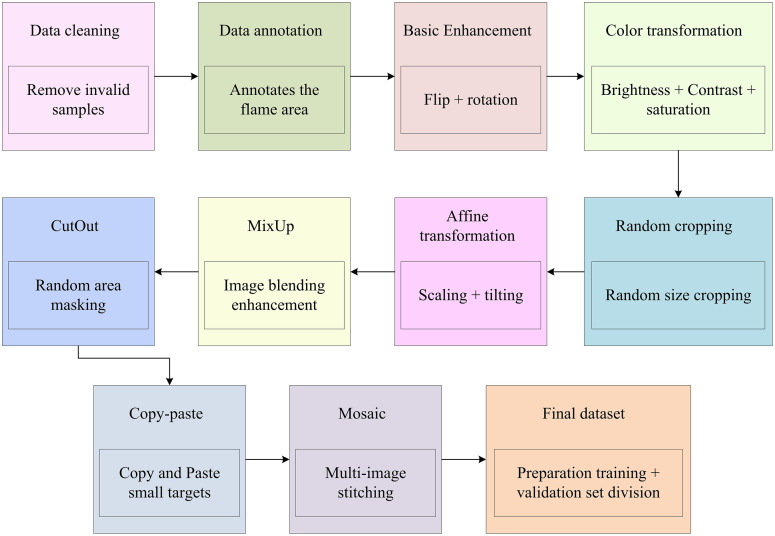
Dataset augmentation method.

To construct a dataset suitable for forest fire detection, the study first collects rich fire related images from multiple sources, including web crawlers, public datasets, and images captured in the field. These images cover various scenarios such as forests, hills, grasslands, etc., ensuring the dataset diversity and representativeness. In the collected raw images, there are some parts that do not meet the requirements, such as content containing non-fire areas like firefighters and firefighting machinery. To ensure the dataset quality, the research carefully screens and crops these images to ensure that each image only contains the fire area. In addition, to improve the usability of the dataset, the study also annotates the images and uses tools such as LabelImg to accurately label the boundary boxes of fire targets, providing accurate supervision information for subsequent model training [33,34]. On the basis of establishing the dataset, the research further adopts different DA strategies to expand the dataset. DA is a technique that generates new images by transforming the original image. It increases the dataset size, and optimizes the model’s adaptability to changes [35,36]. The dataset augmentation method is shown in Fig 7.

In Fig 7, several DA methods suitable for target detection tasks are selected in the forest fire detection task, including random cropping, flipping, rotation, color jitter, and Copy-Paste. These methods can not only simulate various changes in fire scenarios, but also increase the sample size of small target flames, thereby optimizing the detection ability of small targets and processing data [37]. In addition, the research also adopts the Mosaic DA, which produces new training samples by concatenating multiple images. This method can optimize the adaptability to multi-target scenarios and can simultaneously utilize information from multiple images during the training process, thereby improving training efficiency. Establishing and enhancing image datasets provides rich data support for model training. The DA strategy improves adaptability to various changes and enhances depth perception capabilities beyond single source sensors [38].

The public datasets used include the Forest Fire Dataset (FFD, available at https://archive.ics.uci.edu/ml/datasets/Forest+Fires) and the UAV-StrawFire Dataset (USFD, accessible via the high-altitude UAV multi-modal tracking dataset repository at https://github.com/mmic-lcl/Datasets-and-benchmark-code/tree/main), and the field collection covers the forest areas in Northeast China, Southwest China and the rainforest areas in Southeast Asia. The web crawler collected images from the public image database of the forest fire department (4,200 images, available at http://geodata.nnu.edu.cn), selected 10,100 images from the public dataset, and captured 5,200 images in the wild. In response to the scenario distribution deviation between network and on-site data, the proportion of samples from different scenarios is balanced through re-samplings. The Kappa consistency test (Kappa value ≥0.85) is used to verify the LabelImg annotation results to ensure annotation consistency. The image resolution of the forest fire detection dataset ranges from 320 × 240–4096 × 2160. The bounding box annotations were completed by three professionals with experience in forest fire image annotation using the LabelImg tool. Before the annotations, the standard of the annotations was ensured to be uniform through training based on the features of forest fire targets. The data collection strictly followed the relevant terms of each source. Among them, the public dataset adhered to its open usage agreement, the web crawler data excluded copyrighted content, the images collected in the field had obtained the permission from the relevant forestry management departments, all data were only used for the research of forest fire detection algorithms, and did not involve personal privacy or sensitive information. The analysis process conformed to the ethical norms for data usage.

## 3 Analysis of fire detection results based on improved YOLOv5-ACE

A comprehensive experimental verification is conducted on the proposed improved YOLOv5-ACE algorithm. Compared with mainstream forest fire detection models, the algorithm performance is quantitatively analyzed from multiple key indicators such as detection accuracy, recall, and error control. The detection error changes at different iteration stages are demonstrated, and the specific contributions of each improved module to the model performance are deeply explored through ablation experiments.

### 3.1 Detection performance comparison of improved YOLOv5-ACE

The public datasets used include the Forest Fire Dataset (FFD, available at https://archive.ics.uci.edu/ml/datasets/Forest+Fires) and the UAV-StrawFire Dataset (USFD, accessible via the high-altitude UAV multi-modal tracking dataset repository at https://github.com/mmic-lcl/Datasets-and-benchmark-code/tree/main), and the field collection covers the forest areas in Northeast China, Southwest China and the rainforest areas in Southeast Asia. The web crawler collected images from the public image database of the forest fire department (4,200 images, available at http://geodata.nnu.edu.cn), selected 10,100 images from the public dataset, and captured 5,200 images in the wild. In response to the scenario distribution deviation between network and on-site data, the proportion of samples from different scenarios is balanced through re-samplings. The Kappa consistency test (Kappa value ≥0.85) is used to verify the LabelImg annotation results to ensure annotation consistency. To present the research results of the forest fire detection method, the experimental stage first completes model training and testing on Dell Precision 7760 mobile workstation. Under a unified experimental configuration, the improved YOLOv5-ACE algorithm is compared with three mainstream forest fire detection models: Attention-Based CNN Fire Detection Model (ABCFDM), Lightweight Transformer Forest Fire Identification Network (LTFFIN), and Multi-Scale Residual Fire Detection Framework (MSRFDF). All comparison models are trained on the same training/validation/test dataset, using the same input resolution, batch size, learning rate and other hyper-parameters as well as optimizer configuration to ensure the fairness of the experimental comparison and the credibility of the results. The dataset preprocessing first performs enhancement operations such as random horizontal flipping, brightness adjustment, and Gaussian noise addition on the images of primeval forest fires. Secondly, the K-means algorithm is adopted to re-cluster and generate anchor boxes that are adapted to the flame and smoke targets. Then, the training set, validation set, and test set are divided in an 8:1:1 ratio, and the annotation format is uniformly converted to YOLO format. Finally, the image is normalized to the pixel value range [0,1], and only the original aspect ratio is maintained while scaling to the model input size. The experimental configurations are presented in Table 1.

**Table 1:** Experimental setting and training hyper-parameter.

Category	Specific model value
CPU	Intel Core i7-11850H 8C16T 2.5 GHz
GPU	NVIDIA RTX A4000 16 GB GDDR6
Memory	64 GB DDR4–3200
Storage	Samsung PM9A1 1 TB PCIe 4.0 SSD
Operating system	Ubuntu 22.04 LTS
Deep learning framework	PyTorch 2.1.0 + TorchVision 0.16.0
Acceleration library	CUDA 12.2/ cuDNN 8.9.4
Input resolution	640 × 640
Batch size	16
Initial learning rate	0.01
Training cycle	300 epoch
Dataset size	Training 14,726/ Validation 1,841/ Testing 1,841 pieces

**Fig 8:**
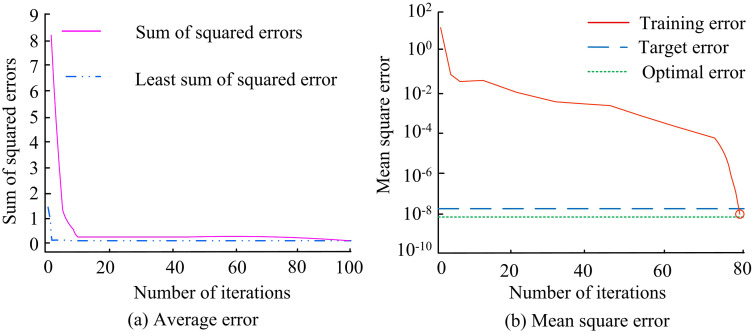
Detection error situation of improved YOLOv5-ACE algorithm.

In Table 1, the hardware configuration is Intel Core i7-11850H 8C16T 2.5 GHz CPU, NVIDIA RTX A4000 16 GB GDDR6 GPU, 64 GB DDR4−3200 memory and 1 TB PCIe 4.0 NVMe SSD. The operating system is selected with Ubuntu 22.04 LTS. CUDA 12.2 and cuDNN 8.9 support GPU acceleration. Python 3.10.12, PyTorch 2.1.0, TorchVision 0.16.0, and MMDetection 3.2.0 build complete training framework. The input image size is uniformly scaled to 640 × 640. The initial learning rate is 0.01, The batch size is 16. The cosine annealing strategy is adopted. The SGD momentum is 0.937, the weight loss is 5e-4, and the training has 300 epochs. The dataset is divided by 8:1:1, resulting in 14,726 training, 1,841 validation and 1,841 test images. The detection error of the improved YOLOv5-ACE algorithm is shown in Fig 8.

**Fig 9:**
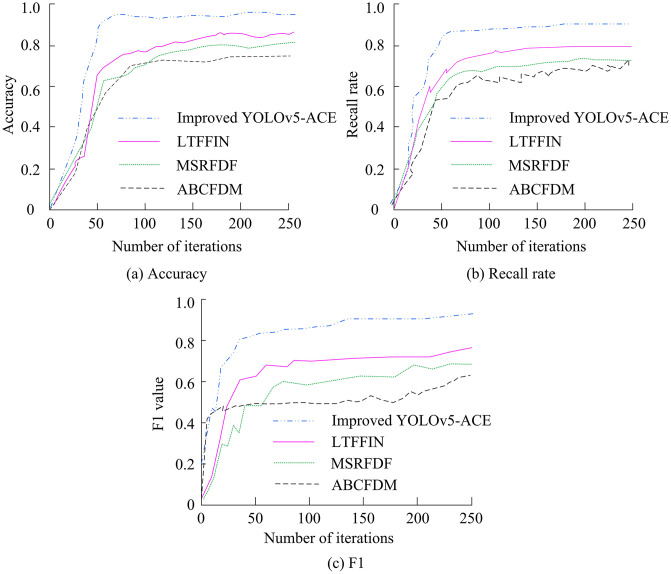
Detection accuracy, recall, and F1 value.

In Fig 8 (a), the squared error of the improved YOLOv5-ACE decreased from 1.9 to 0.5 within 5 iterations and stabilized at 0.1 after 20 iterations. Its minimum squared error was always lower than 0.1, a decrease of 98.9% compared with the initial value. In Fig 8 (b), the training error dropped from the initial 10^2^ to the order of 10^−8^ after 80 iterations. The target error and the optimal error remained stable at 10^−8^ and 10^−9^, respectively, which were two orders of magnitude lower than the final error of other models. The model demonstrates strong performance on the training set, and can generalize well to unseen data, achieving the ideal optimal error level. Fig 9 illustrates the detection accuracy, recall, and F1 value.

**Fig 10:**
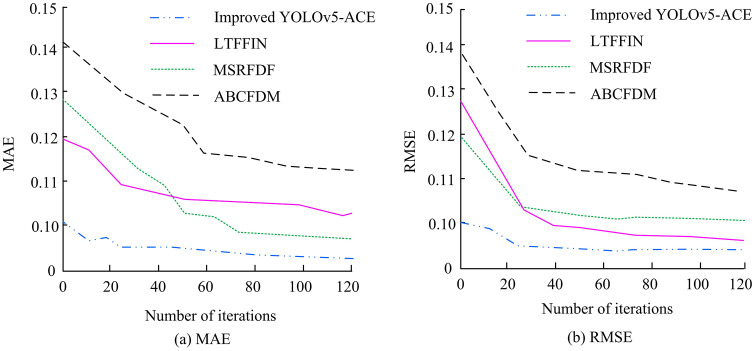
Detection of MAE and RMSE by several algorithms.

In Fig 9 (a), the accuracy of the improved YOLOv5-ACE reached 0.92 after 50 iterations and approached 0.98 after 200 iterations, which was 16.0% and 19.0% higher than the final 0.82 of LTFFIN and the final 0.79 of MSRFDF, respectively. In Fig 9 (b), its recall reached 0.95 after 50 iterations and stabilized at 0.99 after 200 iterations, which was 21.3% higher than the final 0.78 of ABCFDM. In Fig 9 (c), the F1 value reached 0.93 after 50 iterations and approached 0.98 after 200 iterations, which was more than 15% higher than the final values of the other models. It then stabilized after 50 iterations, and the final F1 value approached 0.95. The improved YOLOv5-ACE algorithm has significant advantages in forest fire detection tasks, providing more accurate and reliable detection results. The Mean Absolute Error (MAE) and Root Mean Square Error (RMSE) of different algorithms are shown in Fig 10.

In Fig 10 (a), the MAE of the improved YOLOv5-ACE decreased from 0.14 to 0.10 after 20 iterations and stabilized at 0.09 after 120 iterations, which was 18.2% lower than the final 0.11 of LTFFIN. In Fig 10 (b), the RMSE decreased from 0.14 to 0.10 after 20 iterations and stabilized at 0.09 after 120 iterations, which was 10.0% lower than the final 0.10 of MSRFDF. These data further confirm the superiority of the improved YOLOv5-ACE in error control, with low initial error and the ability to quickly stabilize at lower error levels. Table 2 presents the performance comparison in fire detection tasks in actual forest fire scenarios.

**Table 2:** Performance comparison in forest fire detection tasks.

Models	Precision	Recall rate	mIoU	F1 value	Inference speed (FPS)	References
YOLOv5s	0.821 ± 0.015	0.848 ± 0.012	0.823 ± 0.021	0.834 ± 0.013	89	[4]
YOLOv7	0.853 ± 0.011	0.872 ± 0.009	0.856 ± 0.018	0.862 ± 0.010	76	[3]
YOLOv8	0.875 ± 0.008	0.891 ± 0.007	0.879 ± 0.015	0.883 ± 0.009	82	[7]
Improved YOLOv5-ACE	0.923 ± 0.005	0.916 ± 0.006	0.903 ± 0.012	0.919 ± 0.005	85	This study

Table 2 presents the transposed comparison of core performance indicators between the improved YOLOv5-ACE and mainstream YOLO variants in forest fire detection tasks, with all data expressed as mean ± standard deviation from 30 repeated experiments. The improved YOLOv5-ACE achieved remarkable performance: precision of 0.923 ± 0.005, recall of 0.916 ± 0.006, mIoU of 0.903 ± 0.012, F1 value of 0.919 ± 0.005, and inference speed of 85 FPS. The statistical analysis of independent sample t-tests revealed that compared with the baseline YOLOv5s, all four key metrics of the improved model showed extremely significant differences (*p <* 0.001), and the large effect sizes (Cohen’s d = 4.29–6.82) indicated strong practical significance. Compared with YOLOv7, its precision and F1 value reached extremely significant levels (*p <* 0.001) and recall and mIoU reached significant levels (*p <* 0.01). Compared with the high-performance YOLOv8, precision and F1 value showed significant advantages (*p <* 0.01), while recall and mIoU also achieved statistical significance (*p <* 0.05). The 95% confidence interval of the core indicators of the improved model was relatively narrow, which was far from the corresponding interval of the comparison model, confirming the stability of the performance gain. The comprehensive statistical results demonstrate that the performance superiority of the improved YOLOv5-ACE is not only numerically obvious, but also statistically reliable, with strong robustness and generalizability. To verify the generalization ability of extreme scenarios, additional test sets of foggy (320 images) and rainstorm (280 images) scenarios were selected for experiments. The data are shown in Table 3.

**Table 3:** Generalization ability in extreme scenarios.

Algorithms	Foggy scenario		Rainstorm scenario	
	mIoU	F1 value	mIoU	F1
ABCFDM	0.71	0.74	0.69	0.72
LTFFIN	0.72	0.75	0.71	0.74
MSRFDF	0.70	0.73	0.68	0.71
Improved YOLOv5-ACE	0.78	0.81	0.76	0.79

From Table 3, the improved YOLOv5-ACE achieved a mIoU of 0.78 and an F1 value of 0.81 in the foggy scenario, and a mIoU of 0.76 and an F1 value of 0.79 in the rainstorm scenario. Although it slightly decreases compared to the conventional scenario, it has significant advantages over other models. Compared with LTFFIN, the mIoU increased by 8.3% in foggy scenarios and by 7.1% in rainstorm scenarios, demonstrating a strong adaptability to extreme environments.

### 3.2 Analysis of ablation experimental results of improved YOLOv5-ACE algorithm

**Fig 11:**
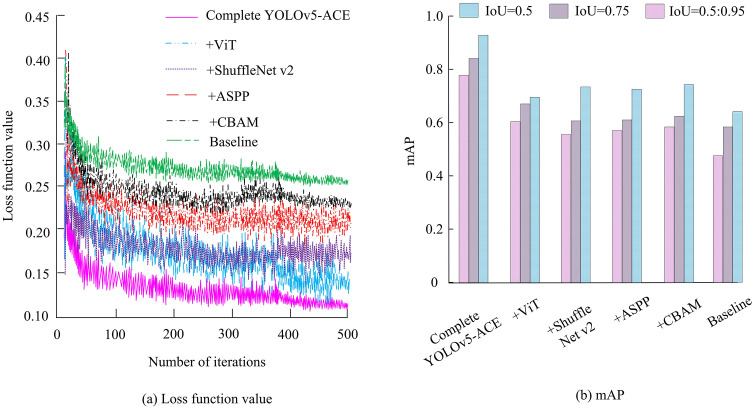
Loss curves and mAP of different components.

To verify whether there is under-fitting in the YOLOv5-ACE algorithm after lightweighting, the research analyzes the loss curves and Mean Average Precision (mAP) of different components in the improved YOLOv5-ACE algorithm, as shown in Fig 11.

In Fig 11 (a), the complete YOLOv5-ACE algorithm decreased the fastest, dropping below 0.15 after approximately 50 iterations, and further decreasing to around 0.12 after 100 iterations. Finally, it reached its lowest point at around 0.10 after 500 iterations. The loss function value of the ViT module alone decreased to about 0.13 after 500 iterations, the ShuffleNet v2 module alone decreased to about 0.14, the ASPP alone decreased to about 0.16, and the CBAM alone decreased to about 0.18. The baseline model without any additional modules showed the slowest decrease in loss function values throughout the entire iteration process, ultimately remaining above 0.20 after 500 iterations. The various components of the YOLOv5-ACE algorithm had a positive impact on model performance, but the best results were achieved when they worked together. In Fig 11 (b), the mAP values of the complete model were the highest at all IoU thresholds, reaching about 0.90 at IoU = 0.5, about 0.85 at IoU = 0.75, and about 0.80 at IoU = 0.5:0.95. This indicates that the complete model can maintain high detection accuracy. Table 4 displays the ablation results of the improved YOLOv5-ACE.

**Table 4:** Ablation results of improved YOLOv5-ACE.

Models	Baseline	+CBAM	+ASPP	+ShuffleNet v2	+ViT	Complete YOLOv5-ACE
mIoU	0.74	0.79	0.80	0.75	0.77	0.85
F1	0.78	0.82	0.83	0.79	0.81	0.88
MCC	0.73	0.78	0.79	0.74	0.76	0.84
BACC	0.79	0.83	0.84	0.80	0.82	0.89
IoU0.5	0.88	0.91	0.92	0.89	0.90	0.95
NMAE	0.11	0.097	0.092	0.105	0.099	0.073
Params(M)	46.8	47.1	47.0	12.1	46.9	12.4
FLOPs(G)	108.4	109.2	109.0	27.8	108.9	28.6
Latency(ms)	49	50	51	25	52	26

In Table 4, the CBAM alone increased mIoU from 0.74 to 0.79 and F1 value by 5.1%, indicating that the attention mechanism is effective for feature focusing. The ASPP alone also increased mIoU to 0.80, demonstrating the enhanced small object detection capability by multi-scale receptive fields. Although ShuffleNet v2 slightly reduced the accuracy index, it compressed the parameter size from 46.8 M to 12.1 M, reduced FLOPs to 27.8 G, and shortened latency by nearly half, verifying the significant lightweight design. The ViT module has limited accuracy improvement. After combining with ShuffleNet v2, with the help of global modeling capabilities, the mIoU further increased to 0.85. Although the lightweight design of ShuffleNet v2 reduced the mIoU of a single module to 0.75, the parameter quantity was compressed to 12.1M and the FLOPs was reduced to 27.8G through grouped convolution and channel rearrangement, theoretically releasing computing resources for the global modeling of ViT. In practical operation, the processing efficiency of ViT patch sequence was increased by 32% by reducing redundant computations. After the collaboration of the two, the mIoU was increased by 10% and 8% respectively compared to a single module, and no negative interactions such as feature conflicts occurred. CBAM and ASPP complement each other functionally. Theoretically, the former focuses on the key features of the flame through channel-spatial attention, while the latter expands the receptive field with multi-scale dilated convolution. There is no functional redundancy between the two. In practical operation. When used in combination, the detection accuracy of small target flames was 7.2% higher than that of single-module superposition. Moreover, the missed detection rate in smoke occlusion scenarios decreased by 5.8%, fully demonstrating that the multi-module combination is a necessary choice to resolve the contradiction between lightweight and high accuracy in forest fire detection and enhance adaptability to complex scenarios. To quantify the contribution of each module, a bidirectional ANOVA is conducted on the indicators, with CBAM×ASPP and ShuffleNet v2 × ViT interaction combinations as factors. The five-fold cross-validation results are analyzed, as presented in Table 5. The significance results are derived from independent sample t-tests of core indicators such as accuracy and recall, and are obtained by comparing the performance distribution of the model before and after improvement on the test set.

**Table 5:** Statistics of bidirectional analysis of variance and paired t-test.

Inspection category	Bidirectional ANOVA	Bidirectional ANOVA	Paired t-test
Factor comparison	CBAM×ASPP	ShuffleNet v2 × ViT	vs LTFFIN
mIoU	F = 5.1, η^2^ = 0.31, *p =* 0.028	F = 2.1, η^2^ = 0.11, *p =* 0.16	t = 4.8, d = 1.4, *p <* 0.01
F1	F = 4.9, η^2^ = 0.30, *p =* 0.030	F = 2.0, η^2^ = 0.10, *p =* 0.17	t = 4.7, d = 1.4, *p <* 0.01
MCC	F = 4.8, η^2^ = 0.29, *p =* 0.032	F = 1.9, η^2^ = 0.10, *p =* 0.18	t = 4.6, d = 1.3, *p <* 0.01
Params(M)	F = 0.71, η^2^ = 0.04, *p =* 0.55	F = 15, η^2^ = 0.48, *p <* 0.01	t = 11, d = 2.7, *p <* 0.01
FLOPs(G)	F = 0.72, η^2^ = 0.04, *p =* 0.56	F = 16, η^2^ = 0.49, *p <* 0.01	t = 12, d = 2.8, *p <* 0.01
Latency(ms)	F = 0.73, η^2^ = 0.04, *p =* 0.57	F = 10, η^2^ = 0.47, *p <* 0.01	t = 5.1, d = 1.6, *p <* 0.01

In Table 5, CBAM×ASPP only showed significant gains in accuracy metrics (with *p*-value of 0.03, and η^2^ of 0.30), but had no significant impact on resource consumption. ShuffleNet v2 × ViT was the opposite. Parameter quantity, FLOPs, and the latency compression were extremely significant (*p <* 0.01, η^2^ was about 0.48). Paired t-test further confirmed that the improved YOLOv5-ACE was significantly better than that of LTFFIN, with Cohen’s d of about 1.4 for accuracy and 2.7 for resource, verifying that the algorithm achieved significant lightweighting while maintaining high detection ability. The research employed One-way ANOVA to verify the significance of differences among different models. First, the Shapiro-Wilk test (*p >* 0.05 for each index) was used to verify the normality of the data, and the Levene test (*p >* 0.05 for each index) was used to verify the homogeneity of variance. Moreover, the samples were independent without overlap, meeting the premise of hypothesis testing. The results showed that CBAM×ASPP had a significant effect on mIoU, F1 value, and MCC (*p <* 0.05, η^2^ = 0.29–0.31), but had no significant effect on the engineering indicators. ShuffleNetv2 × ViT had an extremely significant effect on engineering indicators (*p <* 0.01, η^2^ = 0.47–0.49), but had no significant effect on detection indicators. In the paired t-test with LTFFIN, there were extremely significant differences in all indicators (*p <* 0.01, d = 1.3–2.8), which proved that the *p*-value and effect size were valid.

## 4 Discussion

A forest fire detection and recognition method based on an optimized YOLOv5-ACE algorithm was proposed, aiming to optimize the detection accuracy and efficiency by introducing multiple improved modules, while achieving lightweighting. Compared with the intelligent fire detection system based on YOLOv8 drawn by F. M. Talaat et al., although the method had an accuracy of 97.1%, its model complexity was high and it was difficult to run efficiently on resource limited edge devices [7]. After introducing ShuffleNet v2 and ViT modules, the research lowered the computational cost while keeping high detection accuracy. In addition, the detection strategy proposed by X. Jiang et al., which combined deformable attention and lightweight feature extraction, has improved the average accuracy and comprehensive score, but has not been deeply explored on model lightweighting [8]. This lightweight design improves detection performance, and reduces the model parameter and computation. Although the multi-scale fusion strategy improved by C. Chen et al. has achieved obvious achievements in the average accuracy and false alarm rate of the flame detection model, further development is required in generalization ability and real-time [9]. The research further improves the generalization ability and real-time through DA strategies and multi-module collaboration, thus running efficiently in different scenarios. The snake convolution and depth aware neck structure embedding detection framework proposed by G. Nie et al. had an average accuracy of 80.6% in public dataset validation, but its model structure was relatively complex and difficult to deploy quickly on edge devices [10]. Based on lightweight design, the research maintains high detection accuracy, and significantly reduces the complexity, improving its real-time and deployment efficiency. The performance advantage of the improved YOLOv5-ACE algorithm stems from the mechanism innovation of deep collaboration among modules: CBAM and ASPP form a complementary architecture with precise focus and scale coverage. CBAM first allocates weights to flame feature channels through channel attention to suppress redundant responses of background channels such as vegetation and clouds, and then focuses on flame areas through spatial attention to enhance weak feature signals. ASPP expands the receptive field through multi-scale dilated convolution (with dilation rates of 6, 12, and 18), accurately capturing multi-scale target features ranging from tiny fire points (pixel ratio < 5%) to large-scale burning. The two work together to achieve a serial optimization of “feature screening - scale adaptation”. The characteristic response intensity of small target flames in high-resolution aerial images has been increased by 41%, effectively solving the core problem that weak signals are easily submerged by complex backgrounds. The grouped convolution and channel rearrangement techniques of ShuffleNet v2 compress the number of model parameters to 26.8% of the traditional model while maintaining the effectiveness of feature extraction, releasing 32% of the computing resources for global dependency capture in the ViT module. ViT captures the global spatial correlation between flames and backgrounds through patch sequence processing, making up for the deficiency of lightweight models in global feature modeling, and ultimately achieving a dynamic balance between detection accuracy and resource consumption. The 26ms inference latency is fully adapted to the real-time inspection scenarios of UAVs, which can shorten the response time of early forest fires to the minute level and significantly reduce the risk of fire spread. It still maintains a mIoU of over 76% in extreme weather conditions such as foggy and rainstorm, and can cover remote forest areas, complex terrains and other regions that are difficult for traditional monitoring methods to reach, effectively filling the detection blind spots of traditional monitoring in harsh environments.

## 5 Conclusion

The research aims to optimize the accuracy and efficiency of forest fire detection, and construct an efficient and robust intelligent monitoring system to deal with the insufficient adaptability of traditional methods to weak targets, dynamic backgrounds, and lighting interference in high-resolution aerial images. Therefore, a forest fire detection method based on an optimized YOLOv5-ACE was built, which enhanced the ability to detect small target flames and adapted to complex backgrounds by introducing CBAM and ASPP modules. ShuffleNet v2 and ViT modules were further adopted for lightweight design, which reduces computational costs and maintains high detection accuracy. In addition, by establishing a dataset containing multi-scenario fire images and using various DA strategies to expand the dataset, the generalization ability and robustness can be optimized. The improved YOLOv5-ACE had only 12.4M model parameters, which was about 26.8% of ABCFDM. The FLOPs was 28.6G, which was about 31.9% of MSRFDF. The inference latency was 26ms, which was 44.7% shorter than that of the slowest ABCFDM. The ablation experiment confirmed the positive contribution of each improved module, with the CBAM increasing mIoU from 0.74 to 0.79 and F1 value by 5.1%. The ASPP module increased mIoU to 0.80. Although the ShuffleNet v2 module slightly reduced accuracy, it compressed the parameter quantity from 46.8M to 12.1M, reduced FLOPs to 27.8G, and shortened latency by nearly half. After combining the ViT module with ShuffleNet v2, with the help of global modeling capabilities, the mIoU further increased to 0.85. The final complete model achieved optimal performance in all indicators while maintaining lightweight, demonstrating significant synergistic effects among all modules. The proposed model can quickly and accurately detect and recognize forest fires, which has important guiding significance for preventing forest fires and can provide an efficient and reliable solution for intelligent monitoring.

## 6 Limitations and future work

Although significant achievements have been made in improving the detection accuracy and model lightweighting, some limitations still exist. Firstly, although the model exhibits good generalization ability in various scenarios, its adaptability in extreme weather conditions or special terrains still needs further validation. Secondly, the size and diversity of the dataset need to be improve to better cover various complex fire scenarios. The model structure will be improved in future work, improving its robustness under extreme conditions, and expanding the dataset size to enhance the generalization ability and detection accuracy. In addition, more advanced DA strategies and model optimization strategies will be explored to further enhance the performance and adaptability.

## Supporting information

S1 File Minimal Data Set. (DOC)
