# Supplementary material for: Forest fire detection and recognition method based on improved YOLOv5-ACE algorithm
Source: PLoS One. 2026 Mar 9;21(3):e0343592. doi: 10.1371/journal.pone.0343592 (PMC12970867; doi:10.1371/journal.pone.0343592)
Supplement: S1 File — (DOC) [file pone.0343592.s001.doc]

**Minimal Data Set**

Figure 8 Detection error situation of improved YOLOv5-ACE algorithm

(a) Average error

| Number of Iterations | Sum of Squared Errors | Least Sum of Squared Error |
| --- | --- | --- |
| 0 | 8.020 | 1.200 |
| 10 | 0.500 | 0.009 |
| 20 | 0.100 | 0.007 |
| 30 | 0.050 | 0.005 |
| 40 | 0.030 | 0.004 |
| 50 | 0.020 | 0.003 |
| 60 | 0.010 | 0.002 |
| 70 | 0.005 | 0.001 |
| 80 | 0.001 | 0.001 |

(b) Mean square error

| Number of Iterations | Training Error | Target Error | Optimal Error |
| --- | --- | --- | --- |
| 0 | 1.0×10^2^ | 1.0×10^-8^ | 1.0×10^-8^ |
| 10 | 1.0×10^-1^ | 1.0×10^-8^ | 1.0×10^-8^ |
| 20 | 5.0×10^-3^ | 1.0×10^-8^ | 1.0×10^-8^ |
| 30 | 1.0×10^-4^ | 1.0×10^-8^ | 1.0×10^-8^ |
| 40 | 5.0×10^-6^ | 1.0×10^-8^ | 1.0×10^-8^ |
| 50 | 1.0×10^-6^ | 1.0×10^-8^ | 1.0×10^-8^ |
| 60 | 5.0×10^-7^ | 1.0×10^-8^ | 1.0×10^-8^ |
| 70 | 1.0×10^-7^ | 1.0×10^-8^ | 1.0×10^-8^ |
| 80 | 1.0×10^-8^ | 1.0×10^-8^ | 1.0×10^-8^ |

Figure 9 Detection accuracy, recall, and F1 value

(a) Accuracy

| Number of Iterations | Improved YOLOv5-ACE | LTFFIN | MSRFDF | ABCFDM |
| --- | --- | --- | --- | --- |
| 0 | 0.00 | 0.00 | 0.00 | 0.00 |
| 10 | 0.25 | 0.20 | 0.15 | 0.10 |
| 20 | 0.50 | 0.40 | 0.30 | 0.25 |
| 30 | 0.70 | 0.60 | 0.50 | 0.45 |
| 50 | 0.90 | 0.75 | 0.65 | 0.60 |
| 100 | 0.95 | 0.85 | 0.75 | 0.70 |
| 200 | 0.98 | 0.88 | 0.80 | 0.75 |
| 250 | 0.98 | 0.88 | 0.82 | 0.78 |

(b) Recall rate

| Number of Iterations | Improved YOLOv5-ACE | LTFFIN | MSRFDF | ABCFDM |
| --- | --- | --- | --- | --- |
| 0 | 0.00 | 0.00 | 0.00 | 0.00 |
| 10 | 0.22 | 0.18 | 0.14 | 0.10 |
| 20 | 0.48 | 0.38 | 0.28 | 0.23 |
| 30 | 0.68 | 0.58 | 0.48 | 0.42 |
| 50 | 0.88 | 0.72 | 0.62 | 0.57 |
| 100 | 0.94 | 0.83 | 0.73 | 0.68 |
| 200 | 0.97 | 0.88 | 0.78 | 0.73 |
| 250 | 0.97 | 0.88 | 0.80 | 0.76 |

(c) F1

| Number of Iterations | Improved YOLOv5-ACE | LTFFIN | MSRFDF | ABCFDM |
| --- | --- | --- | --- | --- |
| 0 | 0.00 | 0.00 | 0.00 | 0.00 |
| 10 | 0.23 | 0.19 | 0.15 | 0.11 |
| 20 | 0.49 | 0.39 | 0.29 | 0.24 |
| 30 | 0.69 | 0.59 | 0.49 | 0.43 |
| 50 | 0.89 | 0.73 | 0.63 | 0.58 |
| 100 | 0.93 | 0.84 | 0.74 | 0.69 |
| 200 | 0.94 | 0.89 | 0.76 | 0.74 |
| 250 | 0.94 | 0.89 | 0.76 | 0.75 |

Figure 10 Detection of MAE and RMSE by several algorithms

(a) MAE

| Number of Iterations | Improved YOLOv5-ACE | LTFFIN | MSRFDF | ABCFDM |
| --- | --- | --- | --- | --- |
| 0 | 0.10 | 0.11 | 0.13 | 0.14 |
| 20 | 0.01 | 0.10 | 0.11 | 0.12 |
| 40 | 0.01 | 0.10 | 0.10 | 0.12 |
| 60 | 0.01 | 0.10 | 0.10 | 0.11 |
| 80 | 0.01 | 0.10 | 0.10 | 0.11 |
| 100 | 0.01 | 0.10 | 0.10 | 0.11 |
| 120 | 0.01 | 0.10 | 0.11 | 0.11 |

(b) RMSE

| Number of Iterations | Improved YOLOv5-ACE | LTFFIN | MSRFDF | ABCFDM |
| --- | --- | --- | --- | --- |
| 0 | 0.10 | 0.13 | 0.12 | 0.14 |
| 20 | 0.01 | 0.10 | 0.10 | 0.12 |
| 40 | 0.01 | 0.10 | 0.10 | 0.11 |
| 60 | 0.01 | 0.10 | 0.10 | 0.11 |
| 80 | 0.01 | 0.10 | 0.10 | 0.11 |
| 100 | 0.01 | 0.10 | 0.10 | 0.11 |
| 120 | 0.01 | 0.10 | 0.10 | 0.11 |

Figure 11 Loss curves and mAP of different components

(a) Loss function value

| Number of Iterations | Complete YOLOv5-ACE | +ViT | +ShuffleNet v2 | +ASPP | +CBAM | Baseline |
| --- | --- | --- | --- | --- | --- | --- |
| 0 | 0.40 | 0.40 | 0.40 | 0.40 | 0.40 | 0.40 |
| 100 | 0.15 | 0.20 | 0.25 | 0.25 | 0.25 | 0.30 |
| 200 | 0.12 | 0.18 | 0.23 | 0.23 | 0.23 | 0.28 |
| 300 | 0.11 | 0.17 | 0.22 | 0.22 | 0.22 | 0.27 |
| 400 | 0.11 | 0.16 | 0.21 | 0.20 | 0.21 | 0.26 |
| 500 | 0.11 | 0.15 | 0.20 | 0.21 | 0.25 | 0.26 |

(b) mAP

| Model | mAP@IoU=0.5 | mAP@IoU=0.75 | mAP@IoU=0.5:0.95 |
| --- | --- | --- | --- |
| Complete YOLOv5-ACE | 0.95 | 0.82 | 0.79 |
| +ViT | 0.70 | 0.68 | 0.60 |
| +ShuffleNet v2 | 0.75 | 0.60 | 0.55 |
| +ASPP | 0.75 | 0.60 | 0.58 |
| +CBAM | 0.75 | 0.63 | 0.59 |
| Baseline | 0.65 | 0.58 | 0.48 |
